# Dynamics in Anemia Development and Dysregulation of Iron Homeostasis in Hospitalized Patients with COVID-19

**DOI:** 10.3390/metabo11100653

**Published:** 2021-09-25

**Authors:** Lukas Lanser, Francesco Robert Burkert, Rosa Bellmann-Weiler, Andrea Schroll, Sophie Wildner, Gernot Fritsche, Günter Weiss

**Affiliations:** Department of Internal Medicine II, Innsbruck Medical University, 6020 Innsbruck, Austria; lukas.lanser@i-med.ac.at (L.L.); francesco.burkert@i-med.ac.at (F.R.B.); rosa.bellmann-weiler@i-med.ac.at (R.B.-W.); andrea.schroll@tirol-kliniken.at (A.S.); sophie.wildner@tirol-kliniken.at (S.W.); gernot.fritsche@tirol-kliniken.at (G.F.)

**Keywords:** COVID-19, SARS-CoV-2, disease severity, hyperinflammation, outcome, recovery, anemia, hemoglobin course, iron deficiency, anemia of inflammation

## Abstract

Anemia and disturbances of iron metabolism are frequently encountered in patients with COVID-19 and associated with an adverse clinical course. We retrospectively analyzed 645 consecutive COVID-19 patients hospitalized at the Innsbruck University Hospital. Pre-existing anemia was associated with increased risk for in-hospital death. We further found that the decline in hemoglobin levels during hospital stay is more pronounced in patients with signs of hyperinflammation upon admission, the latter being associated with a nearly two-fold higher risk for new onset anemia within one week. Anemia prevalence increased from 44.3% upon admission to 87.8% in patients who were still hospitalized after two weeks. A more distinct decrease in hemoglobin levels was observed in subjects with severe disease, and new-onset anemia was associated with a higher risk for ICU admission. Transferrin levels decreased within the first week of hospitalization in all patients, however, a continuous decline was observed in subjects who died. Hemoglobin, ferritin, and transferrin levels normalized in a median of 122 days after discharge from hospital. This study uncovers pre-existing anemia as well as low transferrin concentrations as risk factors for mortality in hospitalized COVID-19 patients, whereas new-onset anemia during hospitalization is a risk factor for ICU admission. Anemia and iron disturbances are mainly driven by COVID-19 associated inflammation, and cure from infection results in resolution of anemia and normalization of dysregulated iron homeostasis.

## 1. Introduction

Coronavirus disease 2019 (COVID-19) caused by the novel severe acute respiratory syndrome coronavirus 2 (SARS-CoV-2) emerged as a pandemic, affecting people all around the world [1,2]. Due to the high contagiousness of SARS-CoV-2, COVID-19 challenged health care systems worldwide [3]. While most patients experience only mild symptoms, 10–20% of infected subjects require hospitalization [4]. The severity of the disease can be classified according to the WHO ranging from mild via moderate to severe and critical diseases [5]. Up to 29% of hospitalized COVID-19 patients may develop acute respiratory distress syndrome (ARDS) with need for treatment in an intensive care unit (ICU) and/or ventilation therapy [6].

Several biomarkers predicting morbidity and mortality have been identified to better classify and manage patients upon hospital admission, thus optimizing resource allocation and providing optimal therapy for the patients. Besides male sex, older age or specific genetic factors, several co-morbidities including cardio-cerebrovascular disease, diabetes, respiratory disease, cancer, or chronic kidney disease have been associated with a high morbidity and mortality [4,7,8,9]. In addition, lymphopenia, elevated levels of the immune biomarkers C-reactive protein (CRP), interleukin 6 (IL-6), neopterin or ferritin, low levels of the iron transfer protein transferrin or testosterone deficiency and anemia were shown to be associated with an adverse outcome [10,11,12,13,14]. It has been recently demonstrated that inflammatory biomarkers strongly increase in patients with severe COVID-19 disease and are associated with escalation of respiratory support and survival [11,14]. Erythropoiesis and iron metabolism are strongly influenced by immune activation [15]. Between 56.0% and 68.8% of anemic patients admitted to the hospital presented with anemia of inflammation (AI), which is characterized by increased concentrations of the iron storage protein ferritin together with a reduced circulating iron availability [12,16]. In addition, autoimmune hemolytic anemia was shown to occur occasionally in patients with COVID-19 [17]. Because iron availability as well as erythropoietic factors affect immune function but also infection outcome via controlling the delivery of iron to microbes [18,19,20,21,22], this analysis aimed to investigate the longitudinal association of hemoglobin, iron metabolism, disease severity and survival in hospitalized COVID-19 patients.

## 2. Results

### 2.1. Patients’ Characteristics

We retrospectively analyzed 645 consecutive patients (391 men, 254 women) with polymerase chain reaction (PCR) confirmed SARS-CoV-2 infection and need for in hospital treatment. The median age was 69 years (54–79 years) and similar in men and women (67 vs. 71 years, *p* = 0.331). According to the WHO disease severity scale, more than two thirds of these patients presented with mild or moderate disease (n = 432, 67.0%), while 117 patients had severe or critical disease (18.1%), and 96 patients died during hospital stay (14.9%). In accordance with the literature, men more often presented with severe disease (22.0% vs. 12.2%) or died (17.6% vs. 10.6%), while women more often presented with mild disease (77.2% vs. 60.4%, *p* < 0.001). Patients’ characteristics upon hospital admission within the WHO COVID ordinal scale are depicted in Table 1.

### 2.2. Anemia and Iron Deficiency upon Hospital Admission

About one third of the patients (n = 221, 34.3%) presented with anemia upon hospital admission. Most anemic patients presented with mild (n = 124, 56.1%) to moderate anemia (n = 92, 41.6%), while only 5 patients (2.3%) had a severe anemia with hemoglobin levels <80 g/L. Parameters of iron metabolism were available from 549 patients (85.1%) upon hospital admission. Out of these patients, 417 presented with functional iron deficiency (ID) (76.0%), 36 with absolute ID (6.6%) and 96 had no ID (17.5%). Absolute ID was more prevalent in women compared to men (13.4% vs. 2.1%, *p* < 0.001), while functional ID was equally distributed between men and women (76.9% vs. 74.5%). According to the iron status, anemia of inflammation (AI) was the most frequent etiology, accounting for 143 cases of anemic patients (64.7%), while 12 patients had AI + iron deficiency anemia (IDA) (5.4%), 4 patients had IDA (1.8%) and 31 patients presented with multifactorial anemia (14.0%). However, most patients with multifactorial anemia were normocytic and normochromic (n = 29, 93.5%), presented with elevated median ferritin levels of 1203 μg/L (679–2456 μg/L), CRP levels of 9.36 mg/dL (3.79–21.12 mg/dL) and IL-6 levels of 24.1 ng/L (10.5–101.5 ng/L) as well as decreased transferrin levels of 111 mg/dL (87–137 mg/dL), which are hallmarks of AI. This indicates that most of these patients also had AI, but according to the very low transferrin levels, their transferrin saturation (TSAT) was >20%, which precludes the classical AI definition [15]. 

Hemoglobin levels were available from 249 patients (38.6%) before hospital admission. Interestingly, hemoglobin levels were 129 g/L (114–130 g/L) in a median of 104 days (48–214 days) before hospital admission and therefore did not significantly differ when compared to hemoglobin levels of 130 g/L (113–139 g/L) upon hospital admission for COVID-19 (*p* = 0.303). Pre-existing anemia was predictive of a more than two-fold higher mortality during hospitalization (odds ratio 2.247 (95%CI 1.221–4.135), *p* = 0.009), while the risk of admission to an intensive care unit (ICU) for escalation of respiratory support was not significantly increased (odds ratio 0.674 (95%CI 0.398–1.139), *p* = 0.140). 

### 2.3. Longitudinal Association of Hemoglobin, Hyperinflammation and Disease Severity 

One week (7 ± 1 days) after hospital admission, 484 patients (75.0%) were still hospitalized. In this subgroup of patients, hemoglobin levels declined from 132 g/L (119–144 g/L) to 117 g/L (106–130 g/L, *p* < 0.001) within this time, thereby increasing the prevalence of anemia from 35.3% to 61.0%, which was mainly due to a higher prevalence of multifactorial anemia (38.4% vs. 4.8%). However, most patients with multifactorial anemia after one week were normocytic and normochromic (83.9%) with elevated median ferritin levels of 795 μg/L (440–1419 μg/L), CRP levels of 4.05 mg/dL (1.36–8.02 mg/dL) and IL-6 levels of 19.4 ng/L (7.5–60.6 ng/L), as well as decreased transferrin levels of 131 mg/dL (103–162 mg/dL), again, all hallmarks of AI.

**Figure 1:**
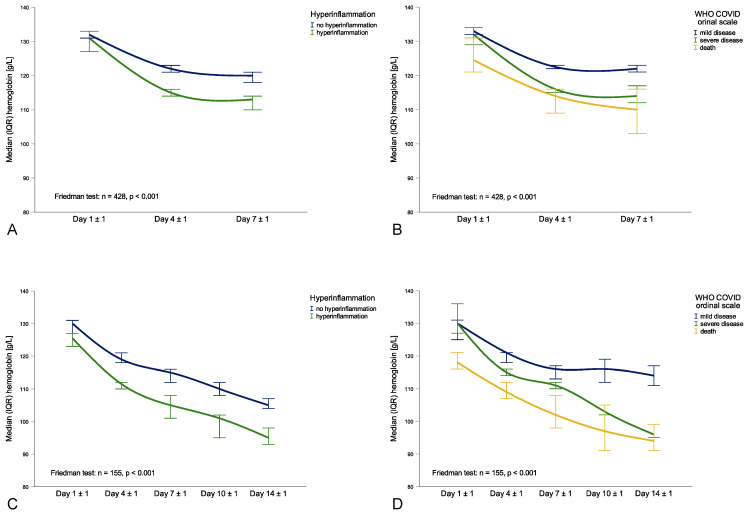
Course of hemoglobin levels during hospital stay within different subgroups: Day 1 ± 1 until Day 7 ± 1 hyperinflammation classification (**A**) and WHO COVID ordinal scale (**B**), Day 1 ± 1 until Day 14 ± 1 for hyperinflammation classification (**C**) and WHO COVID ordinal scale (**D**).

Hyperinflammation upon hospital admission (n = 114, defined as CRP > 15 mg/dL or ferritin > 1500 μg/L [11]) was associated with a significantly higher decline of hemoglobin levels within the first week of hospitalization (131 g/dL to 113 g/dL, *p* < 0.001) compared to patients without hyperinflammation upon hospital admission (n = 370; 133 g/dL to 119 g/dL, *p* < 0.001, Figure 1A). Hyperinflammation upon hospital admission was associated with a nearly two-fold higher risk for new-onset anemia after one week of hospitalization (OR 1.988 (95%CI 1.110–3.559), *p* = 0.021). When dividing patients according to WHO COVID ordinal scale, patients with severe disease (n = 112) had a significantly more profound decline in hemoglobin levels after one week (132 g/L to 114 g/L, *p* < 0.001) when compared to patients with mild disease (n = 308, 133 g/L to 122 g/L, *p* < 0.001), while patients who died during hospital stay already presented with rather low hemoglobin levels at baseline (n = 64, 124 g/L to 110 g/L, *p* < 0.001, Figure 1B). Interestingly, new-onset anemia one week following hospitalization was not associated with a higher mortality (OR 1.430 (95%CI 0.661–3.092), *p* = 0.364) but with a three-fold higher rate of ICU admission (OR 3.115 (95%CI 1.894–5.123), *p* < 0.001).

Two weeks (14 ± 1 days) after hospital admission, 185 patients (28.7%) were still hospitalized. In this subgroup of patients, median hemoglobin levels declined from 129 g/L (117–144 g/L) at baseline to 113 g/L (99–125 g/L) after one week to 103 g/L (90–116 g/L, *p* < 0.001) after two weeks, increasing the prevalence of anemia in this group of subjects from 44.3% to 76.2% to 87.6%. This was due to an increasing prevalence of multifactorial anemia (8.6% to 40.5% to 53.0%), yet most patients with multifactorial anemia after two weeks were normocytic and normochromic (81.6%) with elevated median ferritin levels of 721 μg/L (356–1383 μg/L), CRP levels of 4.07 mg/dL (1.98–9.69 mg/dL) and IL-6 levels of 40.6 ng/L (11.4–75.0 ng/L) as well as decreased transferrin levels of 125 mg/dL (95–150 mg/dL), all hallmarks of AI. Patients with hyperinflammation upon hospital admission (n = 59) had a greater decline in hemoglobin levels from 127 g/dL (111–144 g/dL) at baseline to 107 g/dL (94–119 g/dL) after one week and to 95 g/dL (84–110 g/dL) after two weeks (*p* < 0.001) when compared to patients without hyperinflammation (n = 126; 131 g/dL to 116 g/dL to 106 g/dL, *p* < 0.001). Interestingly, this distinction was especially seen in the first week of hospitalization, while hemoglobin levels further declined similarly between patients with or without hyperinflammation (Figure 1C). When dividing patients according to WHO COVID ordinal scale, patients with severe disease (n = 73) had a significantly more pronounced decline in hemoglobin levels from 130 g/L at baseline to 111 g/L after one week to 96 g/L after two weeks (*p* < 0.001) when compared to patients with mild disease (n = 78, 131 g/L to 117 g/L to 113 g/L, *p* < 0.001), while patients who died during hospitalization already presented with lower hemoglobin levels at baseline (n = 34, 118 g/L to 101 g/L to 94 g/L, *p* < 0.001, Figure 1D).

### 2.4. Longitudinal Association of Ferritin, Transferrin, Hyperinflammation and Disease Severity 

**Figure 2:**
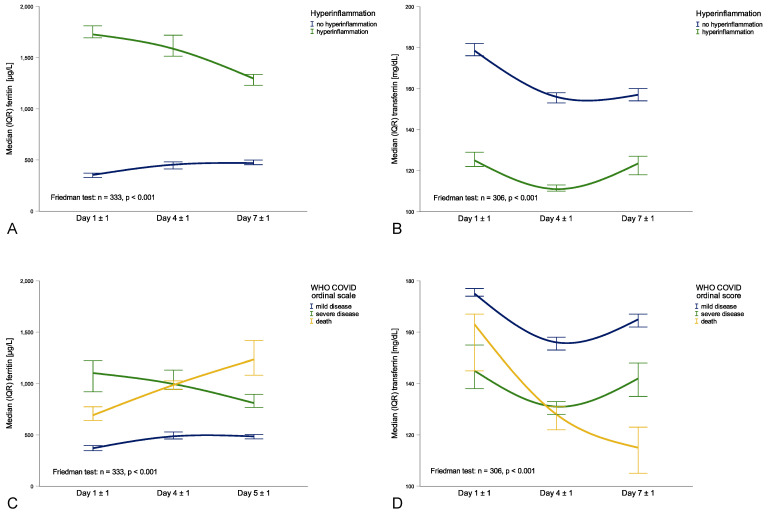
Course of ferritin and transferrin levels during the first week of hospital stay within different subgroups: transferrin and hyperinflammation classification (**A**) or WHO COVID ordinal scale (**B**), ferritin and hyperinflammation classification (**C**) or WHO COVID ordinal scale (**D**).

In patients still hospitalized after one week with available parameters of iron metabolism (n = 426), the increase in ferritin levels from 465 μg/L (248–1103 μg/L) at baseline to 609 μg/L (312–1182 μg/L) after one week was not significant (*p* = 0.705), while transferrin levels significantly decreased from 170 mg/dL (137–202 mg/dL) at baseline to 138 mg/dL (107–167 mg/dL) after one week (*p* < 0.001). Interestingly, patients with hyperinflammation upon hospital admission had a decline in ferritin levels (1738 μg/L to 1318 μg/L, *p* < 0.001, Figure 2A), while transferrin levels almost remained the same (130 mg/dL to 124 mg/dL, *p* = 0.180, Figure 2B). Contrarily, in patients without hyperinflammation upon hospital admission, ferritin levels slightly increased (338 μg/L to 481 μg/L, *p* < 0.001, Figure 2A), while transferrin levels decreased (181 mg/dL to 155 mg/dL, *p* < 0.001, Figure 2B). When dividing patients according to WHO COVID ordinal scale, patients with severe disease had a decline in ferritin levels from 1103 μg/L to 810 μg/L (*p* = 0.036), while ferritin levels did not significantly increase in patients with mild disease (from 360 μg/L to 490 μg/L; *p* = 0.830) but significantly increased in those who died during hospital stay (664 μg/L to 1235 μg/L, *p* < 0.001, Figure 2C). In addition, transferrin levels had a similar course in patients with severe and mild disease during the first week with a short decrease on day 4 ± 1 before rising again on day 7 ± 1, yet transferrin levels continuously decreased in patients who died during hospital stay (Figure 2D).

### 2.5. Normalization of Hemoglobin and Iron Levels after Discharge from Hospital

Follow-up data after discharge from hospital was available from 175 patients (27.1%). In this subgroup of patients, hemoglobin levels increased to 137 g/L (121–148 g/L) over a median of 122 days (101–153 days) after discharge from hospital. When dividing patients according to the WHO disease severity scale, patients with mild disease (n = 131) had a decline in hemoglobin from 133 g/L (118–141 g/L) at baseline to 118 g/L (101–129 g/L) after two weeks of hospitalization, followed by a normalization to 136 g/L (118–146 g/L) in a median of 123 days after discharge from hospital (*p* < 0.001). In patients with severe disease (n = 44), hemoglobin levels declined from 129 g/L (119–144 g/L) at baseline to 95 g/L (88–103 g/L) after two weeks in hospital before increasing again to 146 g/L (128–151 g/L) after a median of 121 days after discharge from hospital (*p* < 0.001). In addition, in patients with hyperinflammation (n = 45), hemoglobin levels declined from 134 g/L (118–145 g/L) at baseline to 95 g/L (83–107) after two weeks before they increased to 146 g/L (129–157 g/L) in a median of 121 days (102–159 days) after discharge from hospital (*p* < 0.001). Finally, ferritin levels decreased to 142 μg/L (64–269 μg/L) and transferrin levels increased to 253 mg/dL (231–283 mg/dL) in a median of 122 days after discharge from hospital. This was also found within the WHO COVID ordinal scale as well as the hyperinflammation classification.

## 3. Discussion

Our data provide several lines of new evidence. First, we have shown that pre-existing anemia is associated with an increased risk of death in hospitalized patients suffering from COVID-19. Second, anemia upon admission is also associated with an increased risk of death, confirming previous data from our group in a smaller patient cohort [12]. Third, hemoglobin levels declined during hospitalization, which was associated with higher frequency of anemia, specifically of anemia of inflammation, reaching a prevalence of up to 87.8% in subjects hospitalized for at least two weeks. Importantly, patients developing anemia were more likely to be admitted to the ICU. Fourth, after discharge, hemoglobin levels normalized in most patients within a median observation period of four months. 

The decline in hemoglobin and the development of anemia during hospitalization is linked to immune activation and cytokine-mediated alterations of iron homeostasis, being hallmarks of anemia of inflammation [15]. Hemoglobin levels remained almost unchanged in patients without hyperinflammation, while they rapidly decreased in patients with hyperinflammation. We also demonstrated that hemoglobin levels were almost concurrent before hospital admission, in the subgroup of patients with available hemoglobin levels within one year before hospital admission. This suggests that deterioration of the clinical situation with further enhanced immune activation affects hematopoiesis, resulting in the development of anemia over time. Several pro-inflammatory cytokines, especially those produced by the T-helper cell type 1 (Th1) immune response, cause disturbances in iron homeostasis and affect erythropoiesis [15,23]. Interferon gamma (IFN-γ), Tumor necrosis factor alpha (TNF-α) and IL-6 increase iron uptake into macrophages of the reticuloendothelial system [24]. Simultaneously, the iron-regulator protein hepcidin, whose synthesis in the liver is stimulated by IL-6, blocks iron release from macrophages, and reduces iron absorption in the intestine [23]. Expression of the iron storage protein ferritin is increased within macrophages due to the accumulation of iron but also based on cytokine mediated induction of ferritin gene expression [25,26,27], which results in iron binding and prevention of toxic radical formation. Accordingly, ferritin levels were significantly higher in patients with hyperinflammation and associated with disease severity. In a subgroup of patients with available hepcidin concentrations upon hospital admission, we found a significant positive correlation of hepcidin, ferritin and IL-6 concentrations (results not shown), which was similar to results from a smaller Italian cohort [28]. In addition, higher circulating ferritin levels also reflect macrophage activation, which is undermined by a positive association of ferritin with neopterin levels, a pteridine produced by macrophages upon IFN-γ stimulation [10]. Finally, circulating ferritin originates from macrophages, further confirming the association between ferritin levels, advanced inflammation and macrophage activation, the latter being a predictor for a poor outcome from COVID-19 [11,29].

Together with this decreased iron availability for erythropoiesis, IFN-γ, TNF-α and IL-1 directly inhibit the production of erythropoietin in the kidney and impair the differentiation and proliferation of erythroid progenitor cells in the bone marrow [30,31,32]. Finally, the quickest acting inflammation-related mechanism causing a reduction in circulating erythrocytes is the upregulation of erythrophagocytosis by hepatic and especially splenic macrophages, thus decreasing the erythrocyte half-life [15]. This would be in line with increased expression of pro-inflammatory cytokines such as IFN-γ or TNF-α in severe courses of COVID-19 [10,29], as both cytokines may affect erythropoiesis or erythrocyte half-life [33,34]. Thus, we demonstrated that hyperinflammation upon hospital admission was associated with a more pronounced decline in hemoglobin levels in the course of hospitalization, which is in agreement with the notion that the severity of AI is linked to the severity of the underlying disease [1]. Accordingly, recent studies suggest a strong association of anemia with more advanced inflammation in patients with COVID-19 [12,16]. However, hospitalization goes along with repeated blood sampling or occult gastro-intestinal bleeding, which may aggravate anemia especially when erythropoiesis is impaired. In a small cohort study involving 24 ICU patients with COVID-19 it was demonstrated that repeated routine blood sampling accounts for about one third of the total patients’ red blood cell mass decrease during ICU admission [35]. Moreover, in-hospital treatment can contain comprehensive infusion therapy, which has a diluting effect on hemoglobin levels, especially in the ICU, where eventually compromised renal function may further affect erythropoiesis via reduced erythropoietin formation and increased circulating hepcidin levels [36,37,38].

However, most patients with SARS-CoV-2 infection presented with normocytic and normochromic anemia and elevated ferritin levels, together with reduced transferrin concentrations, which are hallmarks of anemia of inflammation [39]. Beside upregulation of ferritin expression, transferrin expression is negatively regulated by several cytokines [20,40,41]. We observed a continuous decline in transferrin levels in hospitalized patients specifically in those severely affected by the disease. As transferrin and serum iron concentrations are the basis for TSAT calculations, low transferrin levels may result in TSAT > 20%, which shows the limitations of the anemia of inflammation definition [15].

Anemia upon hospital admission was already demonstrated to be associated with an adverse outcome in patients with COVID-19 [12,42,43], however, whether or not pre-existing anemia is already a risk factor for disease severity could not be demonstrated until now. Herein we demonstrate in the subgroup of patients with available hemoglobin levels before hospitalization, that pre-existing anemia prior to SARS-CoV-2 infection was associated with an increased in hospital mortality from COVID-19. Since we have only limited information on the causes of anemia in such patients, our observation points to the crucial clinical need to evaluate anemia in any patient, to identify the underlying causes and to treat anemia properly [44,45]. Contrarily, new onset of anemia after hospital admission was not associated with an increased in-hospital mortality but a three-fold higher risk for ICU admission. This suggests that new onset anemia strongly reflects inflammation-related disease progression with the hyperinflammatory syndrome causing respiratory failure and need for further respiratory treatment. Finally, we demonstrated that infection related anemia and dysregulated iron homeostasis, which are observed in the case of COVID-19, normalize after a certain period in most patients. 

### Limitations

We retrospectively analyzed 645 consecutive patients with PCR-proven SARS-CoV-2 infection and need for in-hospital treatment in the region of Tyrol. Unfortunately, parameters of iron metabolism were not available from all patients initially included in the study, which resulted in a smaller sample size when analyzing these parameters. Moreover, hemoglobin levels were not available from all hospitalized patients at every time point during in-hospital stay, which represents a certain bias when interpreting the figures. In addition, we did not have medical records from patients after discharge, specifically regarding whether they received any treatment for anemia after hospital discharge. Finally, regional socio-economic conditions must be taken into consideration when interpreting the results, which is why the results do not allow unrestricted generalization to patients with COVID-19 all over the world.

In conclusion, our results point to the important liaison of iron homeostasis, anemia development and outcome for patients with severe COVID-19 and emphasizes the role of pre-existing anemia and dynamic changes of hemoglobin and iron metabolism parameters for the course and risk prediction of the disease. In most cases, anemia is based on inflammation-driven disturbances in iron homeostasis, reduced erythrocyte half-life and cytokine-mediated inhibitory effects of erythropoiesis. However, anemia and low circulating iron as well as increased tissue iron retention may directly affect the course, either by promoting tissue hypoxia and need for oxygen delivery, by contributing to lung injury or by affecting iron access to specific pathogens, thereby increasing the risk of secondary infections [46,47,48,49]. Thus, in deep analysis of iron homeostasis and anemia in the setting of infection and development of specific intervention strategies to combat anemia, disturbed iron homeostasis, tissue hypoxia and iron delivery to pathogens are important research areas to further the insights into this complex network, resulting in improved therapies for severe infection. 

## 4. Materials and Methods

### 4.1. Study Population

We retrospectively analyzed data of 645 consecutive patients with COVID-19, who were hospitalized at the Innsbruck University Hospital between 25 February 2020, and 13 March 2021. All patients were hospitalized because of polymerase-chain-reaction (PCR)-proven SARS-CoV-2 infection and the need for in-hospital medical care. Fatal events, intensive care unit (ICU) admission, and need for invasive or non-invasive ventilation of all patients were recorded during the patients’ hospital stay. Information about patients’ vital status was obtained from the local clinical information system. The study conformed to the principles of the Declaration of Helsinki and was approved by the ethics committee of the Medical University of Innsbruck (ethical vote: 1167/2020, approved 24 July 2020).

### 4.2. Labotratory Measurements

Blood samples were taken from all patients upon hospital admission and during their hospital stay as part of their routine clinical care and analyzed with fully automated tests in the central laboratory of the Innsbruck University Hospital, which undergoes regular internal and external quality controls and evaluations. Laboratory parameters were extracted from the local clinical information system and summarized to day 1 ± 1, day 4 ± 1, day 7 ± 1, day 10 ± 1 and day 13 ± 1. We also collected laboratory parameters up to one month prior to hospital admission and three months after patients were discharged from hospital where available.

### 4.3. Classifications

We defined anemia according to the World Health Organization (WHO) as hemoglobin (Hb) < 130 g/L in men and < 120 g/L in women. Anemia was further classified into severe anemia (Hb < 80 g/L), moderate anemia (Hb 80–109 g/L) and mild anemia (Hb 110–129 g/L in men and 110–119 g/L in women) [50]. Patients with anemia were also classified according to parameters of iron metabolism into those with AI (TSAT < 20% in combination with ferritin > 100 µg/L), IDA (TSAT < 20% in combination with ferritin < 30 µg/L), combined AI and IDA (TSAT < 20% in combination with ferritin 30–100 µg/L) and multifactorial anemia (TSAT > 20%). ID was defined as TSAT < 20% either in combination with ferritin ≤ 100 µg/L, termed absolute ID, or in combination with ferritin > 100 µg/L, termed functional ID [15,44]. Anemia was further classified into microcytic (MCV < 80 fL), normocytic (80–100 fL) and macrocytic (>100 fL), as well as hypochromic (MCHC < 28 pg), normochromic (28–35 pg) and hyperchromic (>35 pg).

Patients’ disease severity was classified according to the WHO disease severity scale: (1) mild disease defined as need for hospitalization without oxygen therapy or oxygen by mask or nasal prongs, (2) severe disease defined as need for non-invasive ventilation, high-flow oxygen, intubation, mechanical ventilation or need for additional organ support (pressors, extracorporeal membrane oxygenation) and (3) death [5]. Finally, hyperinflammation was defined as either CRP > 15 mg/dL or ferritin > 1500 µg/L upon hospital admission [11].

### 4.4. Statistical Analysis

Parameters are depicted as n (%) or medians (25th, 75th percentile) since Gaussian distribution was not given (tested by Shapiro–Wilk test). Mann–Whitney-U test, Kruskal–Wallis test or Pearson chi-square tests were performed to test for significant differences between two or more unrelated groups. Sign-test or Friedman test were used to test for significant differences between two or more related samples. Spearman rank correlation test was used to correlate continuous variables. All tests were two tailed and p-values < 0.05 were regarded as statistically significant. Statistical analysis was performed using SPSS Statistics Version 27.0 for Macintosh (IBM Corporation, Armonk, NY, USA).

## 5. Conclusions

This study demonstrates that in hospitalized patients with COVID-19, hyperinflammation upon hospital admission is associated with new-onset anemia within one week. New-onset anemia is further associated with increased risk of ICU admission, while pre-existing anemia prior to hospital admission is a risk factor for death from COVID-19 in the case of hospitalization. Moreover, while ferritin levels continuously increase and transferrin levels continuously decrease in patients who died during hospital stay, they recover similarly in patients with mild and severe disease. Finally, hemoglobin, ferritin and transferrin levels normalized in a median of 122 days after discharge from hospital.

## Figures and Tables

**Table 1:** Baseline Characteristics of the Total Cohort and Separated for WHO COVID Ordinal Scale.

Characteristic	Total Cohort	Mild Disease	Severe Disease	Death	Sign.
	n = 645	n = 432	n = 117	n = 96	
	Median (IQR)	Median (IQR)	Median (IQR)	Median (IQR)	*p*-Value
Demographic and Clinical Characteristics					
Age [years]	69 (54–79)	68 (53–78)	63 (56–71)	80 (75–88)	<0.001
BMI [kg/m^2^]	26.3 (23.5–29.7)	26.0 (23.5–29.4)	28.4 (24.4–31.4)	24.4 (22.3–27.7)	0.003
Temperature [C]	38.0 (37.2–38.7)	37.8 (37.1–38.6)	38.5 (37.5–39.1)	38.3 (37.6–39.0)	<0.001
SpO_2_ [%]	90 (86–93)	91 (88–93)	88 (84–91)	86 (80–91)	<0.001
O_2_ requirement [L]	2 (0–5)	2 (0–4)	6 (3–8)	6 (2–12)	<0.001
Duration symptoms start until hospitalization [days]	6 (3–9)	6 (3–9)	7 (4–10)	4 (2–8)	0.008
Duration hospitalstay [days]	10 (7–15)	9 (6–12)	19 (12–33)	10 (5–23)	<0.001
Laboratory Findings					
Cholesterol [mg/dL]	125 (102–159)	131 (106–162)	120 (93–151)	116 (96–135)	0.007
LDL [mg/dL]	73 (50–100)	78 (53–105)	68 (50–99)	54 (41–76)	<0.001
HDL [mg/dL]	34 (26–44)	35 (27–46)	30 (22–36)	28 (21–39)	<0.001
Triglycerides [mg/dL]	103 (81–132)	100 (79–128)	110 (92–138)	113 (85–148)	0.017
HbA1c [%]	6.1 (5.7–6.6)	6.0 (5.7–6.5)	6.4 (6.0–6.8)	6.3 (5.7–6.6)	<0.001
Creatinine [mg/dL]	0.97 (0.79–1.26)	0.94 (0.78–1.17)	0.99 (0.81–1.21)	1.20 (0.91–1.88)	<0.001
CRP [mg/dL]	5.06 (2.03–10.17)	3.81 (1.42–7.51)	10.71 (5.78–16.84)	7.61 (3.34–11.92)	<0.001
IL-6 [ng/L]	35.8 (15.6–78.2)	28.8 (11.9–58.7)	50.5 (22.0–120.3)	87.3 (28.7–186.5)	<0.001
Leukocytes [G/L]	5.9 (4.5–8.2)	5.5 (4.3–7.0)	7.4 (5.2–10.1)	6.9 (5.1–11.6)	<0.001
Lymphocytes [G/L]	0.89 (0.63–1.35)	1.02 (0.69–1.46)	0.74 (0.55–1.02)	0.72 (0.41–1.00)	<0.001
Hemoglobin [g/L]	133 (119–144)	134 (122–145)	133 (119–144)	123 (105–144)	0.004
Hematocrit [L/L]	0.384 (0.347–0.417)	0.387 (0.353–0.417)	0.377 (0.345–0.419)	0.362 (0.315–0.411)	0.013
Thrombocytes [g/L]	181 (147–242)	180 (146–238)	196 (155–278)	167 (134–237)	0.015
MCH [pg]	30.2 (28.9–31.3)	30.2 (28.8–31.2)	30.3 (29.2–31.1)	30.1 (29.1–32.0)	0.582
MCV [fL]	87.0 (83.8–90.4)	86.8 (83.7–89.8)	86.9 (84.3–89.8)	88.9 (84.5–92.5)	0.010
MCHC [g/L]	345 (336–353)	345 (338–352)	347 (339–355)	338 (327–350)	<0.001
Iron [µmol/L]	4.8 (3.5–6.9)	5.0 (3.6–7.1)	4.3 (3.1–6.2)	4.1 (3.2–6.2)	<0.001
Ferritin [µg/L]	458 (245–1.096)	363 (217–757)	1.103 (436–1.937)	661 (346–1.353)	<0.001
Transferrin [mg/dL]	172 (139–204)	182 (155–214)	144 (121–182)	142 (114–170)	<0.001
TSAT [mg/dL]	11 (8–17)	11 (8–16)	12 (8–19)	12 (8–22)	0.820

Kruskal–Wallis test was used for comparison between the three subgroups. Sign. = Significance; IQR = interquartile range; BMI = body mass index; SpO_2_ = oxygen saturation; O_2_ = oxygen; LDL = low density lipoprotein; HDL = high density lipoprotein; HbA1c = glycated hemoglobin; CRP = C-reactive protein; IL-6 = interleukin 6; MCH = mean corpuscular hemoglobin; MCV = mean corpuscular volume; MCHC = mean corpuscular hemoglobin concentrations; TSAT = transferrin saturation

## Data Availability

The data presented in this study are available on request from the corresponding author. The data are not publicly available due to privacy restrictions.
